# Doping induced dielectric anomaly below the Curie temperature in molecular ferroelectric diisopropylammonium bromide

**DOI:** 10.1098/rsos.181397

**Published:** 2018-11-28

**Authors:** Kaige Gao, Binbin Zhang, Yunqing Cao, Xiaobing Chen

**Affiliations:** 1 College of Physical Science and Technology, Yangzhou University, Jiangsu 225009, People's Republic of China; 2 State Key Laboratory of Solidification Processing and Key Laboratory of Radiation Detection Materials and Devices and School of Materials Science and Engineering, Northwestern Polytechnical University, Xi'an 710072, People's Republic of China

**Keywords:** doping, dielectric anomaly, molecular ferroelectric, diisopropylammonium bromide

## Abstract

A dielectric anomaly induced by doping has been observed at about 340 K in chlorine-doped diisopropylammonium bromide. The dielectric anomaly has a switchable behaviour, which indicates potential applications on switches and sensors. Temperature-dependent Raman spectrum, X-ray diffraction and differential scanning calorimetry do not show any anomaly around the dielectric anomaly temperature, which prove that the dielectric anomaly does not come from structure phase transition and has no specific heat variety. It is assumed that this dielectric anomaly can be attributed to the freezing of ferroelectric domain walls induced by the pinning of point defects.

## 1. Introduction

Piezoelectric materials have been widely applied in the energy conversion, sensing, driving and frequency fields. Ferroelectrics usually feature a switchable spontaneous electric polarization and have good piezoelectric performance [1]. Relaxor-based ferroelectric single crystals, such as Pb(Zn_1/3_Nb_2/3_)O_3_–PbTiO_3_ and Pb(Mg_1/3_Nb_2/3_)O_3_–PbTiO_3_ attract much attention for their large piezoelectric coefficients up to 2500 pC N^−1^ and high subsequent strain levels up to 0.6% [2,3]. With proper doping and phase engineering, the solid solution single crystal of the relaxor ferroelectrics can reach much larger piezoelectric coefficients than that of pure materials and polycrystals. Doping is believed to be responsible for good mechanical properties and piezoelectric of the relaxor-based ferroelectric single crystals. While doping modification of the inorganic ferroelectrics have been widely studied in the past years [2,4–12], there are few researches relevant to the doping of organic ferroelectrics except some doping of organic–inorganic hybrid ferroelectrics [13]. Researches on the doping of organic ferroelectric would help us to understand the organic ferroelectric better and speed up the application of organic ferroelectric materials. It is essential for us to study the effects of doping on organic ferroelectrics in order to modify organic ferroelectrics' mechanical properties and piezoelectricity.

Recently, a series of simple molecular ferroelectrics have been found, such as diisopropylammonium bromide (DIPAB) [14–18], diisopropylammonium perchlorate [19,20], 4-(cyanomethyl)anilinium perchlorate [21], 4-methoxyanilinium tetrafluoroborate 18-crown-6 [22], (4-amino-2-bromopyridinium)(4-amino-2-bromopyridine) tetrafluoroborate [23], pyridin-4-ylmethanaminium perchlorate [24] and so on, which is composed by ammonium cations and acid ions. The organic–inorganic hybrid ferroelectrics also cause much attention for the designable and tunable characteristics of organic and inorganic components. So far, many organic–inorganic hybrid ferroelectrics have been discovered, namely, (pyrrolidinium)MnX_3_ (X = Cl,Br) [25,26], (pyrrolidinium)CdCl_3_ [27], (cyclopentylammonium)CdCl_3_ [28], (3-pyrrolinium)MCl_3_ (M = Mn,Cd) [29,30], (benzylammonium)_2_PbCl_4_ [31] and so on. Therefore, a huge potential in practical applications of molecular ferroelectric can be foreseen in the coming future, when doping of molecular ferroelectrics would be an important part of molecular ferroelectric research.

Diisopropylammonium chloride (DIPAC) [32] and DIPAB [14–16,33] are good organic ferroelectric materials with large spontaneous polarization (8.9 and 23 µC cm^−2^, respectively) and high Curie temperature, T_c_ (440 and 426 K, respectively), which possesses ferroelectric properties comparable to those of BaTiO_3_. Ferroelectric DIPAC and DIPAB have similar crystal structure (P2_1_) and similar structure phase transition (P2_1_ → P2_1_/m). So, the two materials can form solid solution single crystal at any ratio. This is similar to that of relaxor-based ferroelectric single crystals which have large piezoelectric coefficients and large subsequent strain levels. We have studied the chlorine-doped DIPAB (DIPAB-C) single crystal in the earlier work [34]. It is found that the ferroelectric properties, including spontaneous polarization, the phase transition temperatures, the lattice parameters, etc., can be modulated by doping with congeners. Here we will report a dielectric relaxation phase transition induced by the doping of chlorine in DIPAB single crystals. Temperature-dependent Raman spectrum, X-ray diffraction and differential scanning calorimetry (DSC) prove the phase transition does not have structure phase transition and has no specific heat variety. It is assumed that this relaxation process can be attributed to the freezing of ferroelectric domain walls induced by the pinning of point defects. This may help us understand the ferroelectric properties in DIPAB.

## 2. Material and methods

The single crystal of chlorine-doped DIPAB (C_6_H_16_NBr_1−*x*_Cl*_x_*, DIPAB-C) was grown by slow evaporation of methanol solution containing 1 mol diisopropylamine, *x* mol hydrochloric acid and (1 − *x*) mol hydrobromic acid (*x* = 0–1). The element contents were measured using a CHN elemental analyser (Heraeus CHN-O-Rapid). DSC measurements of single crystals were recorded by using a NETZSCH DSC 200F3 in the temperature range of 300–453 K. The complex permittivity was measured using a Tonghui TH2828A LCR meter. Variable temperature powder X-ray diffraction (PXRD) was performed on a Bruker D8 Advance X-ray diffractometer. Raman spectra were taken using a Horiba Jobin Yvon HR800 spectrometer system with a 488 nm laser line from an air-cooled Ar-ion laser.

## 3. Results and discussion

Chlorine content in the single crystal of DIPAC was determined by the carbon mass fraction measured by a combustion method using a CHN elemental analyser [34]. The actual chlorine concentration is a little less than the stoichiometric values due to the faster volatility of hydrochloric acid compared with that of hydrobromic acid. As the DIPAB-C samples have similar properties when changing the doping content, here we just choose C_6_H_16_NBr_0.76_Cl_0.24_ (DIPAB-C1) as an example to analyse in the following.

The structure of ferroelectric DIPAB-C is P2_1_, similar to that of pure DIPAB. But the structure of the as-grown crystal of DIPAB-C is P2_1_2_1_2_1_, which is non-ferroelectric. Room temperature ferroelectric DIPAB-C could be acquired by heating the samples above the first phase transition temperature at about 420 K due to the irreversible phase transition. The following measurements were all performed on the ferroelectric samples.

**Figure 1.**
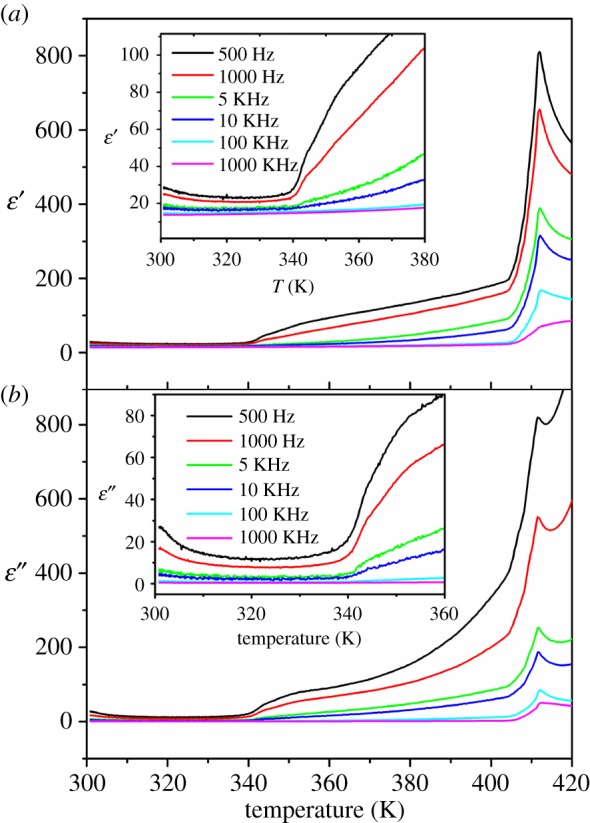
Temperature dependence of (*a*) real part (*ɛ*′) and (*b*) imaginary part (*ɛ*″) of complex dielectric constant of DIPAB-C1 in heating process. Inset: the enlarged view of (*a*) real part (*ɛ*′) and (*b*) imaginary part (*ɛ*″).

The dielectric constant of DIPAB-C1 was measured in the heating cycle with the heating rate of 2 K min^−1^. Figure 1 shows the temperature-dependent complex dielectric constant (*ɛ* = *ɛ*′ − i*ɛ*″, where *ɛ*″ is the imaginary part) at the frequency range from 500 Hz to 1000 KHz. The real part (*ɛ*′) of the complex dielectric is shown in figure 1*a* and the imaginary part (*ɛ*″) is shown in figure 1*b*. Two dielectric anomalies can be seen in figure 1. A very sharp dielectric constant appears at 412 K (T_c_), which belongs to the ferroelectric–paraelectric phase transition. At 412 K, DIPAB-C1 undergoes a structure phase transition from P2_1_ to P2_1_/m. In the vicinity of T_c_, the temperature-dependent dielectric constant along the polar axis follows the Curie–Weiss law of ferroelectric materials parametrized as *ɛ*′ = C/(T − T_0_), as shown by the linear relationship between reciprocal dielectric constant and temperature in electronic supplementary material, figure S2.

It is interesting for us to find another slow step-like dielectric anomaly at about 340 K (T_1_), which is not found in the pure DIPAB and has not been reported in the previous literature. The dielectric anomaly has a switchable behaviour, which indicates potential applications on switches and sensors. Both the real part *ɛ*′ and the imaginary part *ɛ*″ of the complex dielectric display slow step-like dielectric anomaly. When heating across T_1_, the dielectric frequency dispersion has an abrupt increase, characterizing an abrupt increase of relaxation time phase transition. Furthermore, above T_1_, the frequency dispersion of *ɛ*′ and *ɛ*″ enhance with the increase of temperature, indicating the relaxation time decrease with the increasing temperature. Below T_1_, the frequency dispersion keeps almost constant, indicating the relaxation time is independent of temperature. Simply speaking, the relaxation time keeps constant below T_1_, increases abruptly at T_1_ and increases slowly above T_1_.

**Figure 2.**
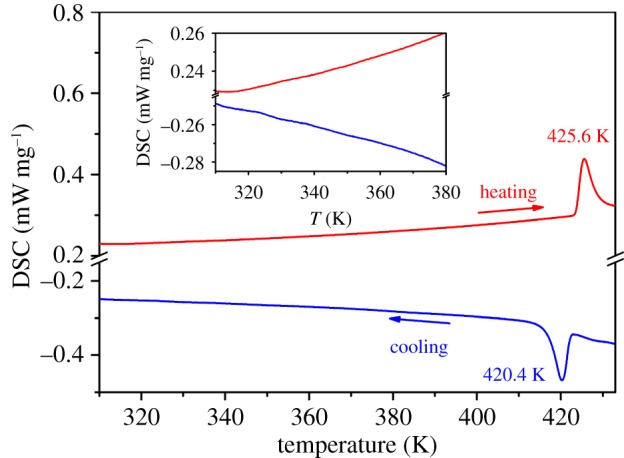
Temperature dependence of DSC curve at a rate of 10 K min^–1^ for the compound. The inset is the enlarged DSC curve from 310 to 380 K.

In order to understand the mechanism of the dielectric anomaly happening at T_1_, DSC measurements were performed. The heating and cooling temperature speed during the DSC measurements are both 10 K min^–1^. The ferroelectric–paraelectric phase could be found easily in figure 2, which displays an endothermic peak at 425.6 K during the heating process and an exothermic peak at 420.4 K during the cooling process. 5 K thermal hysteresis indicates the phase transition is a first-order phase transition. Yet no DSC signal anomaly was found around 340 K, proving the dielectric anomaly at T_1_ has no specific heat variety.

**Figure 3.**
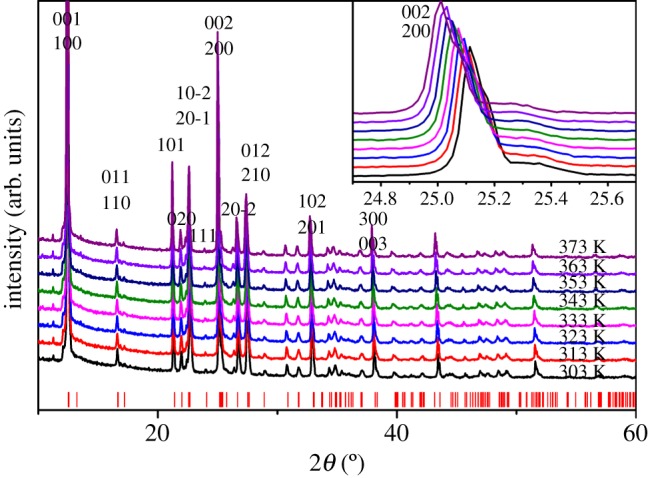
Temperature dependence of PXRD. The inset is the enlarged parts from 24.7° to 25.7°. The short vertical bars indicate the calculated positions of pure DIPAB.

Temperature-dependent PXRD measurements of DIPAB-C1 samples were performed from 303 to 373 K to examine the structure change during the dielectric anomaly happening at T_1_. All of the PXRD patterns have similar diffraction peaks with the peaks shifting uniformly with increasing temperature (figure 3 inset). The PXRD results prove the structure phase transition does not happen at T_1_. Both PXRD and DSC reveal the dielectric anomaly at T_1_ is not structure phase transition and has no specific heat variety. According to the shifts of XRD peak of plane (200) and Bragg equation 2*d*sin*θ* = *n* * *λ*, we can calculate the variance of the plane space *d* along (100) direction against temperature. The calculated coefficient of thermal expansion *α* is 60 × 10^−6^ K^−1^, which is just half of that of polyvinylidene fluoride (PVDF). The large thermal expansion coefficient indicates DIPAB-C1 is as soft as polymer, illustrating DIPAB-C possesses potential electrostriction application [35].

**Figure 4.**
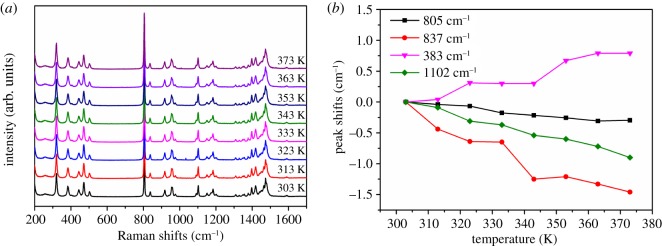
(*a*) Temperature dependence of Raman spectrum. (*b*) The temperature dependence of the Raman peak position shifts with reference to the peak at 303 K.

In order to understand the mechanism and dynamics of the phase transitions, we performed Raman measurements as a function of temperature (figure 4), which is an ideal tool for capturing the dynamics and local structural changes from the viewpoint of lattice vibrations [35]. Figure 4*a* shows the Raman spectra measured from 303 to 373 K. The intensity of the Raman peaks in figure 4*a* do not appear to abruptly change. Some typical peaks' positions shifts according to that of 303 K are shown in figure 4*b*. There are no apparent abrupt shifts at about 340 K. The chosen Raman peak positions almost show a linear variation with temperature. The temperature-dependent Raman spectrum proves the lattice vibrations are not affected by the dielectric anomaly at T_1_. Raman spectrum results deepen the conclusion that the dielectric anomaly does not belong to a structure phase transition.

From DSC, PXRD and Raman measurements, we have reached a conclusion that the dielectric anomaly at T_1_ is not structure phase transition. Then what is responsible for the dielectric anomaly? One reasonable explanation may be the freezing of ferroelectric domains at low temperature induced by the defects in DIPAB-C1. It has been reported that ferroelectric domains in some ferroelectric materials normally froze at low temperature [12,36], which can be attributed to the collective pinning of randomly distributed pinning centre to domain walls. Suppose the ferroelectric domains freeze below T_1_, the relaxation time will be long so that it is difficult to establish relaxation polarization and only instantaneous polarization is in operation. This could easily explain why the dielectric constant hardly changes with temperature and have less frequency dispersion below T_1_. Once the ferroelectric domains unfreeze at high temperature, the relaxation time will decrease with the increase of temperature, which behaves as that the frequency dispersion increase with the temperature increasing.

In addition, when the doping chlorine content *x* is low, the change ratio of the dielectric constant is small around T_1_. Electronic supplementary material, figures S3–S6 show the dielectric constant curves when *x* = 0.024, 0.062, 0.41 and 0.60. The dielectric constant increases at 500 Hz by 7.6%, 25%, 521%, 428% and 160% in the vicinity of T_1_ when *x* = 0.024, 0.062, 0.24, 0.41 and 0.60, respectively. Therefore, the dielectric anomaly is closely related with the doping content. At low doping level, the defects density will increase with the doping content increase. The increased doping level will make freezing process more clear. It is worth noting that defects exist in pure DIPAB single crystals unavoidably, so the freezing process of ferroelectric domains exists unavoidably. This may explain that it is hard for the spontaneous polarization in DIPAB to flip through eternal applied electric field at low temperature.

## 4. Conclusion

A dielectric anomaly was found at 340 K in the chlorine-doped DIPAB, which does not appear in pure DIPAB. Relaxation modes are different near the dielectric anomaly temperature. Above the dielectric anomaly temperature, the dielectric frequency dispersion is larger, and below the dielectric anomaly temperature, the dielectric frequency dispersion is less. DSC, PXRD and Raman spectrum measurements show the dielectric anomaly does not come from structure phase transition and has no specific heat variety. The freezing of ferroelectric domains at low temperature may be responsible for the dielectric anomaly. The dielectric anomaly was induced by doping in DIPAB.

## Supplementary Material

Electronic Supplementary Information

## Data accessibility

Our data are deposited at Dryad Digital Repository: https://doi.org/10.5061/dryad.rs39j28 [37].
