# Supplementary material for: Doping induced dielectric anomaly below the Curie temperature in molecular ferroelectric diisopropylammonium bromide
Source: R Soc Open Sci. 2018 Nov 28;5(11):181397. doi: 10.1098/rsos.181397 (PMC6281938; doi:10.1098/rsos.181397)
Supplement: Electronic Supplementary Information [file rsos181397supp1.docx]

**Electronic Supplementary Information**

**Doping induced dielectric anomaly below the Curie temperature in molecular ferroelectric diisopropylammonium bromide**

Kaige Gao^a*^, Binbin Zhang^b^, Yunqing Cao^a^, Xiaobing Chen^a^

^a^ College of Physical Science and Technology, Yangzhou University, Jiangsu 225009, China

^b^ State Key Laboratory of Solidification Processing & Key Laboratory of Radiation Detection Materials and Devices &School of Materials Science and Engineering, Northwestern Polytechnical University, Xi’an 710072, China

*^
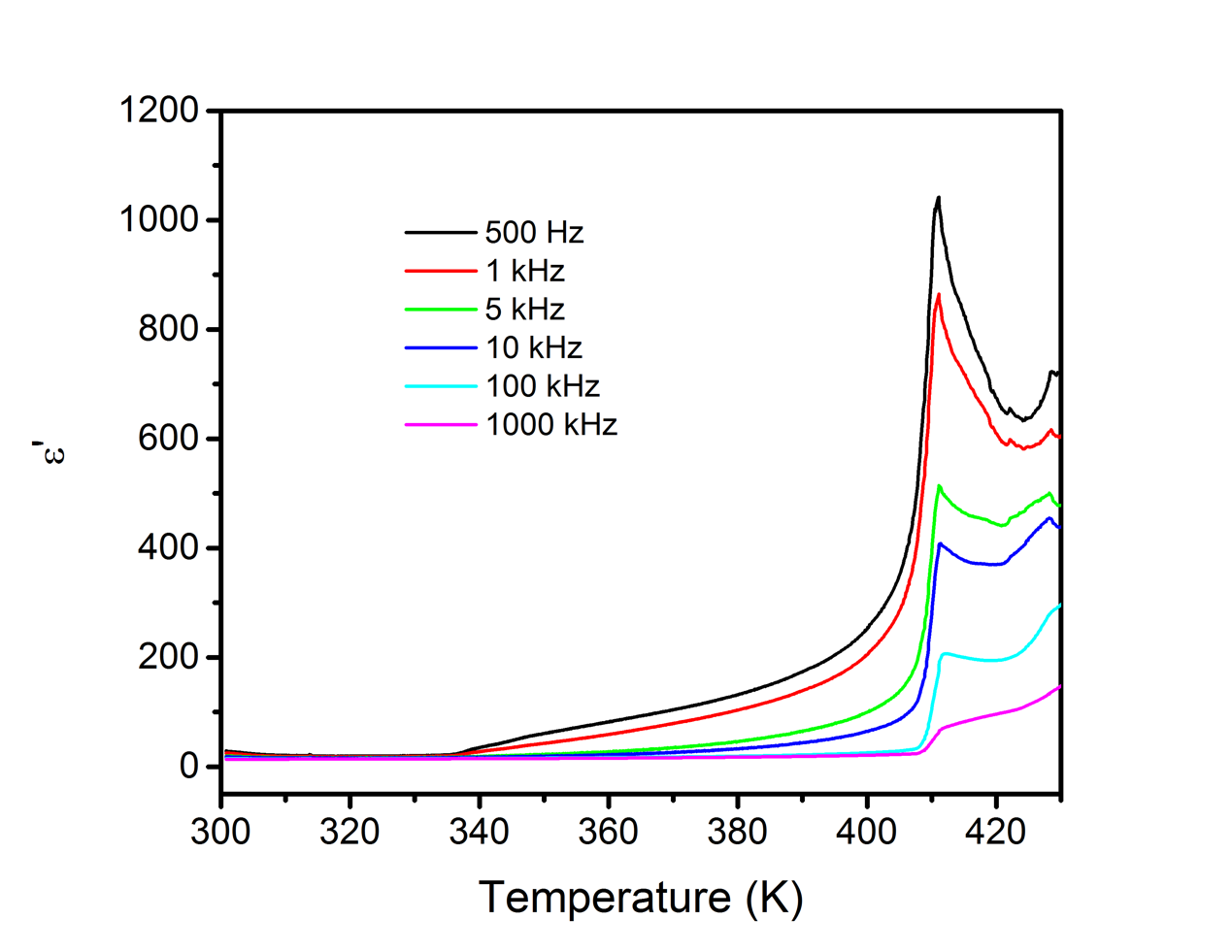
^*

**Figure S1:** The temperature dependent dielectric constant of DIPAB-C1 during the cooling process.


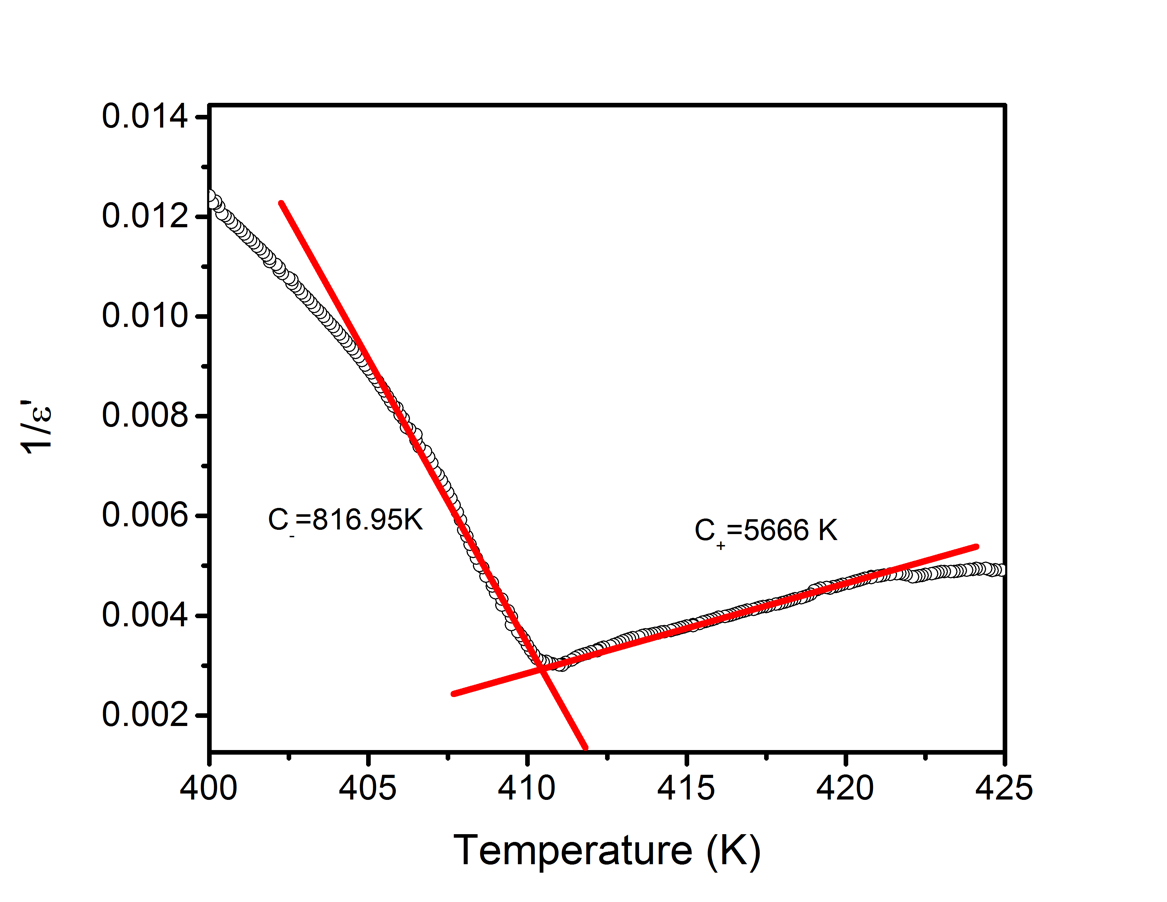


**Figure S2:** 1/ε´ of DIPAB-C1 single crystal sample follows the Curie-Weiss law. The Curie temperature were calculated to be 5666 K and 818.95 K, respectively.


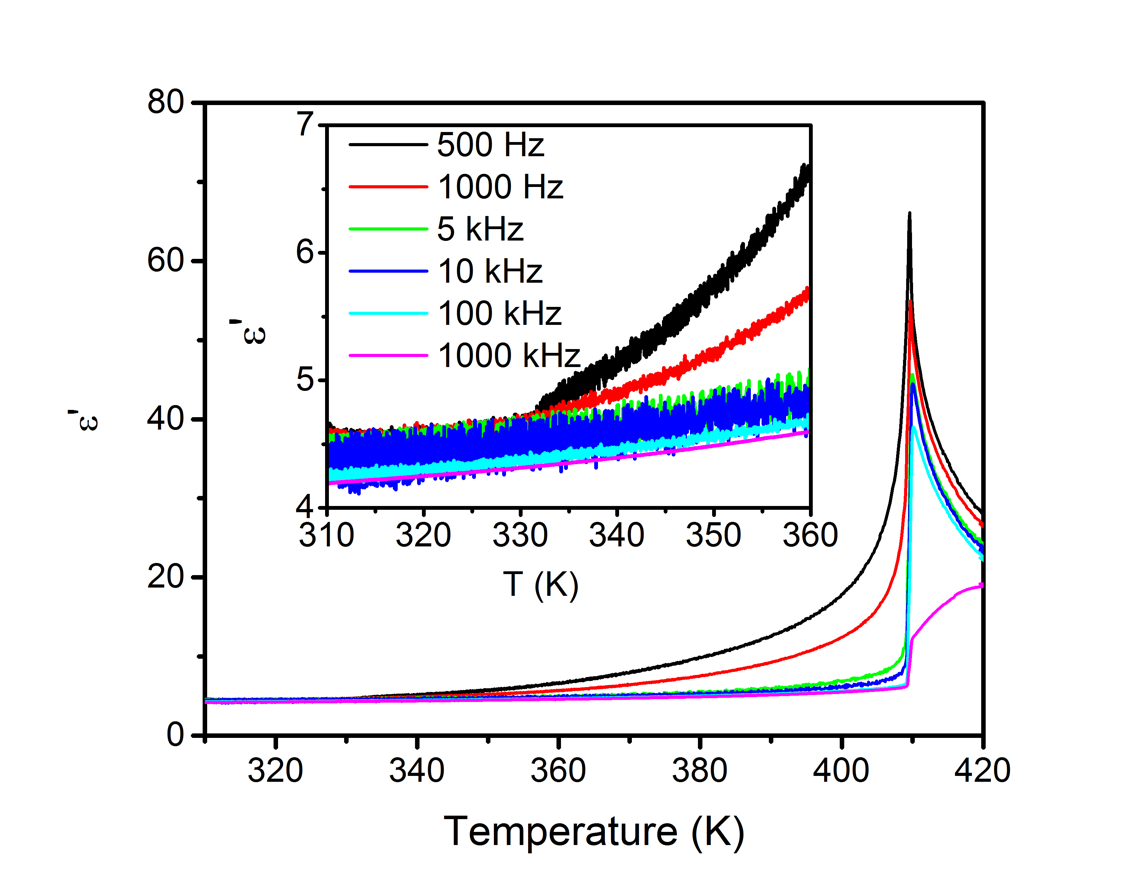


**Figure S3:** The dielectric constant of C_6_H_16_NBr_1-x_Cl_x_ (x=0.024).





**Fig. S4:** The dielectric constant of C_6_H_16_NBr_1-x_Cl_x_ (x=0.062).





**Fig. S5:**The dielectric constant of C_6_H_16_NBr_1-x_Cl_x_ (x=0.41).





**Fig. S6:**The dielectric constant of C_6_H_16_NBr_1-x_Cl_x_ (x=0.60).
